# Retinal phenotype of APOB100 transgenic mice on a Western diet with human-like hyperlipidemia and cholesterol crystals in the retina and choroid

**DOI:** 10.1038/s41684-026-01693-x

**Published:** 2026-02-09

**Authors:** Nicole El-Darzi, Tim F. Dorweiler, Natalia Mast, Julia Busik, Irina A. Pikuleva

**Affiliations:** 1 Department of Ophthalmology and Visual Sciences, Case Western Reserve University, Cleveland, OH USA; 2 Department of Biochemistry and Physiology, University of Oklahoma Health Sciences Center, Oklahoma City, OK USA; 3 Present Address: Department of Surgery, Harvard Medical School, Boston, MA USA

**Keywords:** Macular degeneration, Retina

## Abstract

Drusen and subretinal drusenoid deposits, the pathognomonic lesions for age-related macular degeneration (AMD), are rich in cholesterol. Yet, AMD is not consistently linked to plasma lipids. Here wild-type and human apolipoprotein B100-expressing (APOB100) mice were put on a Western type of diet for 13 months and then assessed for plasma lipid profile, high-density lipoprotein (HDL) heterogeneity, status of intraretinal and choroidal vasculatures, retinal structure, function, levels of cholesterol and other sterols, lipid and cholesterol distribution and expression of cholesterol-related genes. The dietary effects were more pronounced in APOB100 mice, which had human-like hyperlipidemia and different subpopulations of HDL_3_, than in wild-type mice. In addition, the APOB100 retina showed increased cholesterol input from the systemic circulation, higher cholesterol content, more cholesterol crystals, elevated expression of HDL-related genes, lipid accumulation in the retinal pigment epithelium and Bruch’s membrane, and impaired function compared with the wild-type retina. Remarkably, in both genotypes, cholesterol crystals were detected in the choroid, piercing toward Bruch’s membrane and leading to macrophage infiltration. Our data indicate how plasma lipid profile could be linked to AMD and that cholesterol crystals in the choroid should be further investigated as contributors to AMD development and progression.

## Abstract (web summary)

APOB100 mice on a Western diet develop human-like hyperlipidemia, retinal cholesterol buildup, choroidal cholesterol crystals and impaired retinal function, supporting their use for studying lipid contributions to age-related macular degeneration.

## Main

**Fig. 1:**
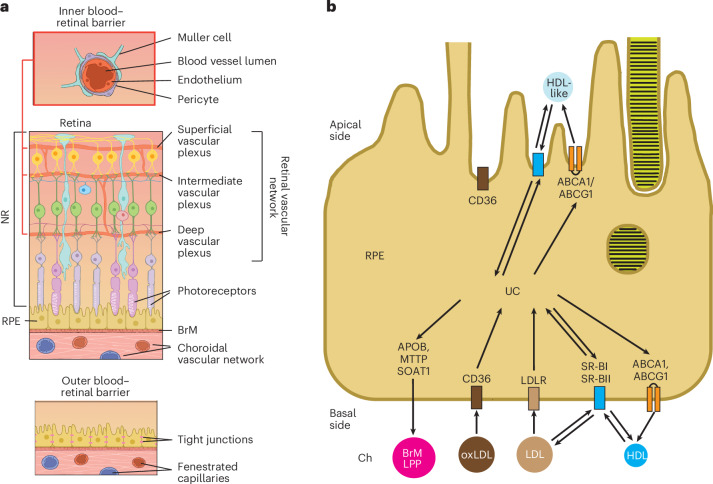
The retina and RPE. **a**, Schematic representation of the retina showing its overall structure, supporting vascular networks and blood–retina barriers. While only three plexi of the retinal vasculature are indicated as having the inner blood–retinal barrier, all blood vessels within the NR have this barrier. **b**, Schematic representation of a RPE cell showing various cholesterol-related proteins and receptors. See the main text for details. Panel **a** was licensed from Carlson Stock Art and is adapted from ref. ^92^ under a Creative Commons license CC BY 4.0. Panel **b** is adapted from ref. ^26^ under a Creative Commons license CC BY 4.0. CD36, cluster of differentiation 36; LDLR, LDL receptor; MTTP, microsomal TG transfer protein; oxLDL, oxidized LDL; SOAT1, sterol *O*-acyl-transferase 1; SR-BI and SR-BII, scavenger receptor class B members I and II, respectively.

The neural retina (NR) is an extension of the brain that initiates the transmission of the visual signal^1^. The NR lines the back of the eye and is composed of different neuron types, which are organized in layers (Fig. 1a). The outermost layer of the NR consists of the photoreceptor cells, which form a complex with the apical side of the underlying monolayer of retinal pigment epithelium (RPE). The RPE’s basal side rests on Bruch’s membrane (BrM), a planar vessel wall, which separates the NR–RPE complex (often called the retina) from the choroid (Ch)^2,3^. The Ch mainly provides blood supply to the RPE and photoreceptors, and the remaining retina is served by the retinal vascular network^4^. The endothelial cells of retinal blood vessels are nonfenestrated and form tight junctions or the inner blood–retinal barrier, which prevents passage of plasma proteins and lipoprotein particles (LPPs) into the NR. Conversely, the blood vessels of the Ch are fenestrated, and plasma macromolecules can reach the RPE, which has tight junctions and forms part of the outer blood–retina barrier. Yet, the RPE has various receptors on both of its sides (Fig. 1b), which mediate the selective exchange between the Ch and RPE as well as between the RPE and NR^4,5^.

Age-related macular degeneration (AMD) is a leading cause of blindness in older individuals of industrialized countries^6^. AMD is a multifactorial disease with age, genetic factors, environment and lifestyle contributing to disease susceptibility and progression^7^. Early AMD stage is characterized by cholesterol-rich deposits accumulated external to the RPE: subretinal drusenoid deposits (SDDs) located apically to the RPE (in the subretinal space between the photoreceptors and RPE) and/or drusen found basolaterally to the RPE in BrM^8–12^. As these extracellular deposits become larger, they ultimately lead to RPE atrophy, photoreceptor degeneration and, in some cases (10–15%), abnormal blood vessel growth (neovascularization) into the retina^13–16^.

Several polymorphisms in the genes (*CETP*, *LIPC*, *APOE* and *ABCA1*) related to high-density lipoprotein (HDL) in the systemic circulation are risk factors for AMD^17^. Nevertheless, numerous studies did not find consistent associations between AMD and plasma lipid profiles^7,18,19^, leading to several explanations. First, plasma lipid profiles can be specific to the disease stage (early, intermediate or late) or disease type (non-neovascular or vascular) as AMD is a heterogeneous disease^19^. Second, AMD could be linked to a specific HDL subclass, as HDL particles are heterogeneous and may differ in their AMD risk-conferring properties^7,18^. Accordingly, small rodent models with blood content similar to that of humans are required to test these explanations and decipher the role of HDL and plasma lipid profile in AMD etiology and progression. Indeed, humans carry most of their blood cholesterol on low-density lipoprotein (LDL), whereas rodents carry it on HDL. Hence, the two species have very different absolute amounts and ratios between their LDL and HDL. In normolipidemic humans, the LDL/HDL ratio should not exceed 2.2 (<100/>45 mg/dL, Table 1)^20^, whereas in C57BL/6J mice and Golden Syrian hamsters, this ratio varies from 0.04 (2.2/51 mg/dL) to 0.09 (5.8/66 mg/dL) and from 0.21 (17/81 mg/dL) to 0.49 (27/55 mg/dL), respectively, depending on the diet^21^. In addition, of the two HDL subclasses (HDL_2_ and HDL_3_), HDL_2_ is the predominant HDL subclass in mice (71%) and hamsters (66%) but is less abundant in humans (19% in men and 30% in women)^22,23^.

**Table 1:** A comparison of plasma and retinal cholesterol levels in humans and different mouse genotypes

Group	Plasma lipids					Retinal TC, nmol/mg protein (F/M)
	TC, mg/dL (F/M)	LDL, mg/dL (F/M)	HDL, mg/dL (F/M)	LDL/HDL (F/M)	TG, mg/dL (F/M)	
Humans	<150	<100	>45	2.2	<150	49 (M)

**ND**						
C57BL/6J mice	106/105	4/2	44/46	0.09/0.04	67/75	47/51
APOB100 mice	102/121^S,G^	25^G^/14^S,G^	28^G^/41^S^	0.89/0.34	79/125	45/46

**WTD**						
C57BL/6J mice	171/333^S,D^	12/30^D^	65/115^S,D^	0.18/0.26	97/89	97^D^/111^S,D^
APOB100 mice	223^D^/520^S,G,D^	88^G,D^/251^S,G,D^	52^D^/93^S,G,D^	1.69/2.70	223^G,D^/194^G,D^	124^G,D^/156^S,G,D^

The values of plasma lipids for humans are recommended by the American Heart Association^20^. Data for mice on ND are from ref. ^26^ and for mice on a WTD are from the present work. Only mean values are shown. Please see Supplementary Fig. 1 for the values in individual mice and significant difference between: ^S^sexes within the same genotype and diet; ^G^genotypes on the same diet; ^D^diets within the same genotype. Data for human retina are taken from ref. ^52^. The ND (Prolab Isopro RMH 3000) contained 0.02% (w/w) cholesterol and 5% (w/w) fat and was given to mice for 11 months from weaning to 12 months of age. The WTD (TD.88137 Envigo) contained 0.15% (w/w) cholesterol and 21% fat (w/w) and was given to mice for 13 months from weaning to 14 months of age. F, female mice; M, male mice.

Previously, we and others characterized the retina of mice with transgenic expression of human apolipoprotein B100 (APOB100 mice)^24–26^ as mice produce only the truncated APOB48 variant^26^. APOB100 mice were fed normal rodent chow (ND) and were compared with wild type (WT), C57BL/6J mice, on the same diet^26^. The transgenic animals had an 8.5-fold (males) and 10-fold (females) increase in their plasma LDL/HDL ratio due to an increase in the LDL content (Table 1), yet did not reach the human LDL/HDL ratios^26^. Moreover, male but not female mice had a 21% increase in the levels of their plasma total cholesterol (TC)^26^. Nevertheless, the retinal content of TC was not changed in both APOB100 sexes (Table 1), although there were lipids deposits in the RPE and BrM^26^ and mouse retinal function was impaired^26^. Our data suggest that a moderate increase in plasma TC and up to a 10-fold rise in the LDL/HDL ratio can be managed in APOB100 mice by the RPE, which produces unique APOB-containing BrM-LPP^27–30^ and cycles plasma cholesterol and lipid excess back to the Ch rather than NR^26^. In the present work, we fed APOB100 and C57BL/6J mice a Western-type diet (WTD), which made the plasma lipid profile of APOB100 mice very similar to that of hyperlipidemic humans, particularly in males. We conducted chorioretinal characterizations of both genotypes and gained insights into why the plasma lipid profile may not always correlate with AMD, while highlighting the importance of preventing systemic hyperlipidemia.

## Results

### Plasma lipid and albumin content

**Fig. 2:**
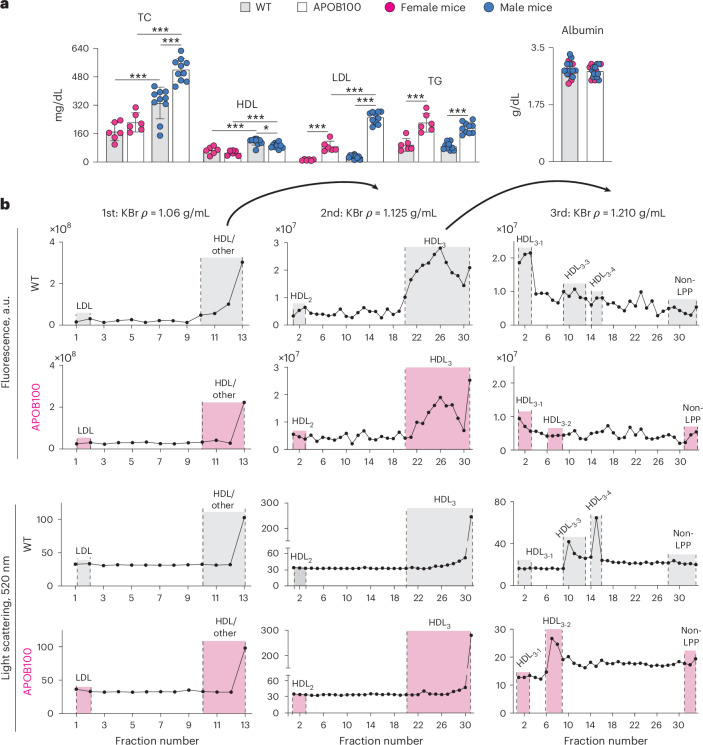
Plasma lipids. **a**, Quantification of fasting plasma lipids. Data represent the mean ± s.d. of the measurements in individual mice (*n* = 5 per each genotype and sex). Data were first assessed for normal distribution by Shapiro–Wilk test and then by one-way ANOVA with Tukey’s multiple comparison test. *P ≤ 0.05, ***P ≤ 0.001. **b**, Isolation of plasma LDL and HDL by three KBr density ultracentrifugations after plasma samples (each 0.2 mL) were pooled from four male mice and subjected to centrifugation at 15,000*g*, 4 °C, for 30 min to remove the chylomicron and light-density LDL fractions. Then, after each ultracentrifugation, 1-mL fractions were collected from the tube top to the tube bottom and measured for ICG fluorescence and light scattering. Fraction pooling for WT and APOB100 mice is indicated by the gray and magenta highlights, respectively. a.u., arbitrary units.

The clinical guidelines for humans suggest that the levels of plasma TC and triglycerides (TG) should be less than 150 mg/dL (ref. ^20^) (Table 1). Both lipids were at higher levels in the WTD-fed APOB100 mice versus WT animals: TC at 223 versus 171 mg/dL in female mice and 520 versus 333 mg/dL in male mice; and TG at 223 versus 97 mg/dL in female mice and 194 versus 89 mg/dL, in male mice (Fig. 2a and Table 1), documenting hyperlipidemia. Also, in humans, the LDL levels are usually higher than those of HDL and should not exceed 100 mg/dL (ref. ^20^) (Table 1). Only in WTD-fed APOB100 mice were the levels of LDL cholesterol (88 and 251 mg/dL in female and male mice, respectively) higher than those of HDL cholesterol (52 and 93 mg/dL in female and male mice, respectively); moreover, only in males did LDL levels exceed those recommended for humans. Similarly, the LDL/HDL ratio in male (2.70) but not female (1.69) APOB100 mice was higher than that (2.2) defined in humans as normolipidemic^20^. Thus, putting WT and APOB100 mice on a WTD for 13 months led to human-like hyperlipidemia only in the APOB100 genotype.

Furthermore, both sexes of the WTD-fed APOB100 mice versus WT mice had substantial increases in the levels of LDL cholesterol (7.3-fold, 88 versus 12 mg/dL in female mice; and 8.4-fold, 252 versus 30 mg/dL in male mice) and TG (2.3-fold, 223 versus 97 mg/dL, in female mice and 2.2-fold, 194 versus 89 mg/dL, in male mice). Yet, only male APOB100 mice had a significant increase in the TC levels versus male WT mice (1.6-fold, 520 versus 333 mg/dL) and a significant decrease in the HDL levels (0.8-fold, 93 versus 115 mg/dL). This is a reflection that, in both genotypes, the plasma effects of WTD were more pronounced in male than female mice (Fig. 2a).

Besides the lipid profile, the plasma of WTD-fed WT and APOB100 mice was analyzed for albumin content and HDL subclasses. This is because cellular efflux of unesterified cholesterol (UC) to HDL and LDL is facilitated by albumin^31–34^ and is higher to HDL_3_ than HDL_2_, as the former contains less cholesterol^35,36^. The albumin levels were the same in WT and APOB100 mice (Fig. 2a), and both genotypes had the canonically floating HDL_2_ and HDL_3_ (designated as HDL_3-1_) after the second and third density ultracentrifugations, respectively (Fig. 2b). Also, both groups had additional peaks on light scattering after the third ultracentrifugation, which were designated according to their increasing density: HDL_3-2_ in the plasma of APOB100 mice, and HDL_3-3_ and HDL_3-4_ in the plasma of WT. Thus, in both genotypes on WTD, the HDL_3_ heterogeneity was increased, but the density of their HDL_3_ subpopulations was different, suggesting that functional properties of these subpopulations could be different as well.

### Retinal sterol profiles

**Fig. 3:**
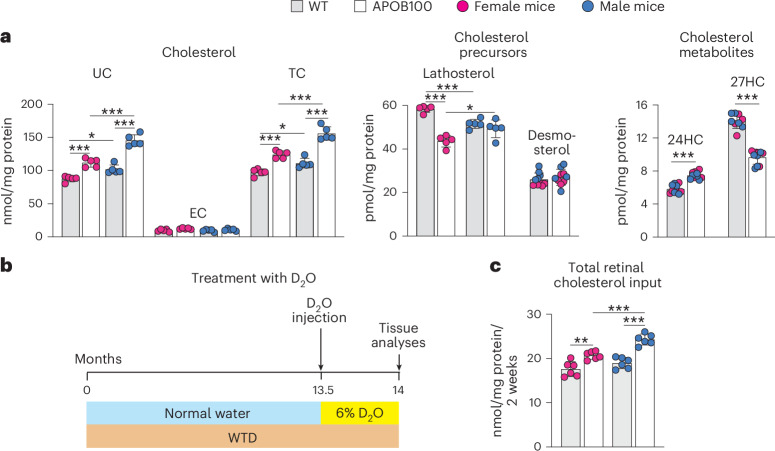
Retinal sterols and cholesterol input. **a**, Quantification of retinal sterols. Data represent the mean ± s.d. of the measurements either in individual retinas (UC, EC, TC, lathosterol and desmosterol: five female (magenta circles) and five male (blue circles) mice per genotype) or in pooled samples (24-hydroxycholesterol (24HC) and 27-hydroxycholesterol (27HC)), each containing two retinas from two different animals of the same genotype and sex (four samples from female and five samples from male mice per metabolite). Data were analyzed by two-way ANOVA with Tukey’s multiple comparisons test. When no statistical significance was found between female and male mice, data for both sexes were combined within the genotype; otherwise, data were presented separately. *P ≤ 0.05, ***P ≤ 0.001. **b**, Experimental design for the quantification of total retinal cholesterol input. **c**, Total retinal cholesterol input. Data represent the mean ± s.d. of the measurements in individual retinas (six female (magenta circles) and six male (blue circles) mice per genotype) and were analyzed by a two-tailed unpaired Student’s *t*-test. **P ≤ 0.01, ***P ≤ 0.001.

The quantifications of retinal sterols were of the three forms of cholesterol (UC, esterified cholesterol (EC) and TC), lathosterol and desmosterol (cholesterol precursors and markers of cholesterol biosynthesis in neurons and astrocytes, respectively^37,38^) and 24-hydroxycholesterol and 27-hydroxycholesterol (cholesterol metabolites generated by CYP46A1 and CYP27A1, respectively^39,40^). In APOB100 mice versus WT mice on WTD, the retinal levels of UC were increased in both sexes, while the EC levels remained unchanged, leading to an increase in the retinal TC levels of 1.40-fold (156 versus 111 nmol/mg protein) in male mice and 1.28-fold (124 versus 97 nmol/mg protein) in female mice (Fig. 3a). Among the cholesterol precursors, there was only a change (decrease) in the lathosterol levels and only in APOB100 female mice (0.76-fold, 44 versus 58 pmol/mg protein), suggesting a compensatory decrease in cholesterol biosynthesis in neurons. Among the cholesterol metabolites, both sexes of APOB100 mice had increased cholesterol elimination via 24-hydroxycholesterol (1.29-fold, 7.5 versus 5.8 pmol/mg protein) but decreased cholesterol elimination via 27-hydroxycholesterol (0.69-fold, 9.6 versus 14 pmol/mg protein).

The steady-state levels of retinal cholesterol represent the balance between the pathways of retinal cholesterol input and output^41^. The former can be measured by putting mice on D_2_O (Fig. 3b) as its [^2^H] atoms could be detected in retinal cholesterol synthesized in situ and obtained from the systemic circulation^42^. In the WTD-fed ABOB100 versus WTD-fed WT animals, the rate of total retinal cholesterol input was increased 1.18-fold in female mice (20.7 versus 17.6 nmol/mg protein/2 weeks) and 1.28-fold (24.3 versus 18.8 nmol/mg protein/2 weeks) in male mice (Fig. 3c), although retinal cholesterol biosynthesis (lathosterol and desmosterol levels; Fig. 3a) was not increased. Collectively, these results suggest that retinal uptake of plasma cholesterol was increased in APOB100 mice of both sexes, leading to elevated retinal TC levels. This input was higher in APOB100 males than in females, probably reflecting their more severe hyperlipidemia and/or reduced integrity of the outer blood–retina barrier, thus enabling more LPPs from the Ch to enter the retina and disturb retinal cholesterol homeostasis.

### Retinal gene expression

**Fig. 4:**
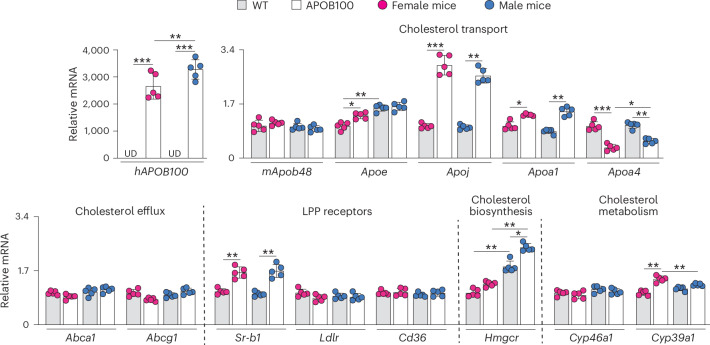
Retinal gene expression. Data represent the mean ± s.d. of the relative mRNA measurements in pooled samples of two retinas from different mice, a total of five samples per genotype and sex. Data were analyzed by two-way ANOVA with Tukey’s multiple comparisons test. *P ≤ 0.05, **P ≤ 0.01, ***P ≤ 0.001. UD, undetectable.

To gain mechanistic insights, retinal expression of the cholesterol-related genes was quantified (Fig. 4). The human *APOB100* expression was 2,666- and 3,200-fold higher than that of mouse *Apob48* in female and male mice, respectively, thus confirming the APOB100 transgenic expression. This result also suggests that lipid processing in human RPE could differ substantially from mouse RPE as *APOB100* expression was under the human promoter^43^, and apolipoprotein B (APOB) is a component of BrM-LPP formed in the RPE to remove excess cholesterol^27–30^. Increases in the expression of apolipoproteins (except *Apoa4*), which putatively form the HDL-like particles in the retina^44^, were observed as well, either in both sexes of APOB100 mice (*Apoj* and *Apoa1*) or only in female mice (*Apoe*) and were supported by increased expression of *Sr-b1*, which binds HDL. However, in both sexes of APOB100 versus WT mice, cellular cholesterol efflux to retinal LPPs did not seem to increase, suggesting that retinal increases in TC (Fig. 3a) were extracellular. Increased expression of *Hmgcr* controlling cholesterol biosynthesis (Fig. 4) was not confirmed by retinal measurements of lathosterol and desmosterol (Fig. 3a), whereas increased expression of *Cyp39a1*, which metabolizes 24-hydroxycholesterol, was consistent with increased expression of this oxysterol in APOB100 mice (Fig. 3a). Thus, collectively, the gene expression measurements suggested an upregulated cholesterol transport on BrM-LPP and retinal HDL-like LPP plus increased cholesterol metabolism by CYP46A1 and CYP39A1, probably reflecting the compensatory changes in the APOB100 versus WT retina in response to increased retinal TC levels. In addition, a lower increase in the *APOB100* expression in female mice than in male mice could in part explain their less severe hyperlipidemia.

### Retinal structure and function

**Fig. 5:**
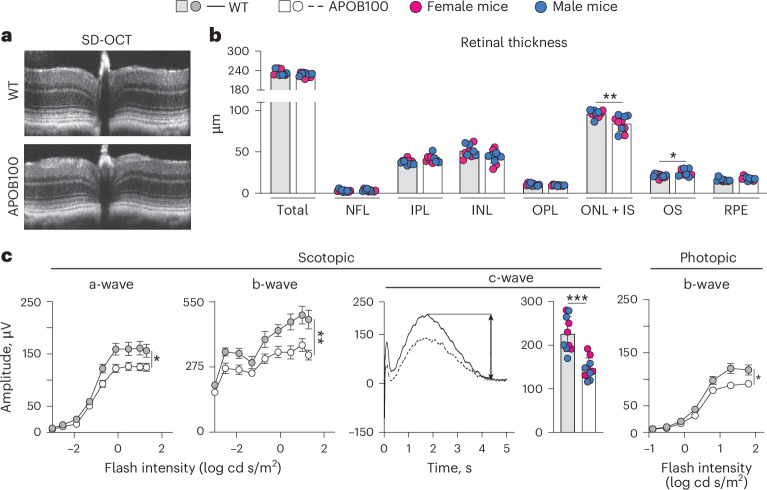
Retinal structure and function. In all experiments, five mice per genotype and sex were used. **a**, Representative images of retinal cross-sections on SD-OCT. **b**, Retinal thickness as assessed by SD-OCT. Data represent the mean ± s.d. of the average layer thickness in both eyes of individual mice and were analyzed by an unpaired nonparametric Mann–Whitney test. *P ≤ 0.05, **P ≤ 0.01. **c**, Retinal ERG responses. a- and b-waves represent the mean ± s.e.m. of the sum of the measurements in both mouse eyes. For the c-wave, both averaged waveforms (left) and the waveform amplitude quantifications in the individual animals (averaged for both eyes, right) are shown. Data were analyzed by two-way ANOVA with Tukey’s multiple comparison test. c-waves represent the mean ± s.d. of the average response in individual mice. Data were analyzed by one-way ANOVA with Tukey’s multiple comparison test. *P ≤ 0.05, **P ≤ 0.01, ***P ≤ 0.001. INL, inner nuclear layer; IPL, inner plexiform layer; IS, photoreceptor inner segments; NFL, nerve fiber layer; ONL, outer nuclear layer; OPL, outer plexiform layer; OS, photoreceptor outer segments.

Structural assessment by spectral domain optical coherence tomography (SD-OCT) suggested no major abnormalities in either WTD-fed WT and WTD-fed APOB100 mice (Fig. 5a) but detected genotype-specific changes in the thickness of several retinal layers (Fig. 5b). Compared with WT mice, both sexes of APOB100 mice had a modest decrease in the thickness of the layer composed of the photoreceptor somas and inner segments (0.88-fold, 96 versus 84 μm) and a modest increase (1.19-fold, 25 versus 21 μm) in the thickness of the layer composed of the photoreceptor outer segments (Fig. 5b). This pattern of changes suggested the photoreceptor degeneration, which, however, was not further investigated.

Functional assessment by the measurements of the electroretinography (ERG) responses (the wave amplitudes) documented a decrease in APOB100 mice versus WT mice in all ERG amplitudes (a-, b- and c-waves under scotopic conditions and b-wave under photopic conditions; Fig. 5c), suggesting a decrease in the overall retinal and RPE function.

### Status of vascular networks

**Fig. 6:**
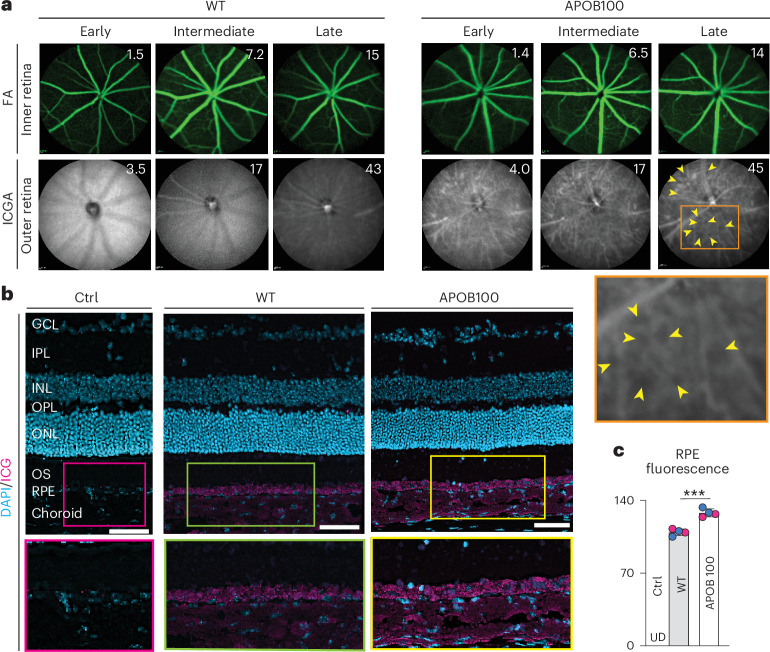
Retinal and Ch vasculatures. **a**, Representative (*n* = 5 per genotype and sex) FA images showing the status of retinal vasculature and ICGA the status of Ch vasculature. Early, intermediate and late angiography phases are shown, with post-injection times (in minutes) indicated in the top right corner. Yellow arrowheads point to the areas of hypofluorescence. **b**, Histological ICG tracking after mice were injected with ICGA and euthanized 13 min post-injection. Representative (*n* = 2 per genotype and sex) retinal cross-sections are shown. Colored rectangles denote enlarged regions. **c**, Quantification of the RPE fluorescence. Data represent the mean ± s.d. of the measurements in individual mice (*n* = 2 per genotype and sex) and were analyzed by a two-tailed unpaired Student’s *t*-test. ***P ≤ 0.001. Ctrl, control; GCL, ganglion cell layer. Scale bars, 50 μm.

Fluorescein angiography (FA) to evaluate the retinal vascular network was apparently normal for both WT and APOB100 mice fed WTD and did not detect any blood vessel leakage on different angiography phases (Fig. 6a). Yet, indocyanine green (ICG) angiography (ICGA) to evaluate Ch showed an apparently homogeneous fluorescence pattern on different angiography phases in the WTD-fed WT mice and a heterogeneous fluorescence pattern in the WTD-fed APOB100 mice. The latter exhibited numerous hypofluorescent spots, which were more clearly visible during the late ICGA phase (Fig. 6a).

Hence, ICG fluorescence was next tracked histologically 13 min after dye injection (Fig. 6b). In both genotypes, the ICG fluorescence was mainly localized to the Ch-RPE region and was more intense in APOB100 than in WT mice (Fig. 6c). In the RPE, the fluorescence was punctate, possibly a reflection of the dye association with the LPPs or vesicles, and seemed to be more heterogeneous in APOB100 than in WT mice, consistent with imaging by angiography. Thus, in vivo imaging and histology tracking of ICG suggested that there may be lipid deposition in the Ch-RPE region of APOB100 mice.

### Chorioretinal lipid distribution

**Fig. 7:**
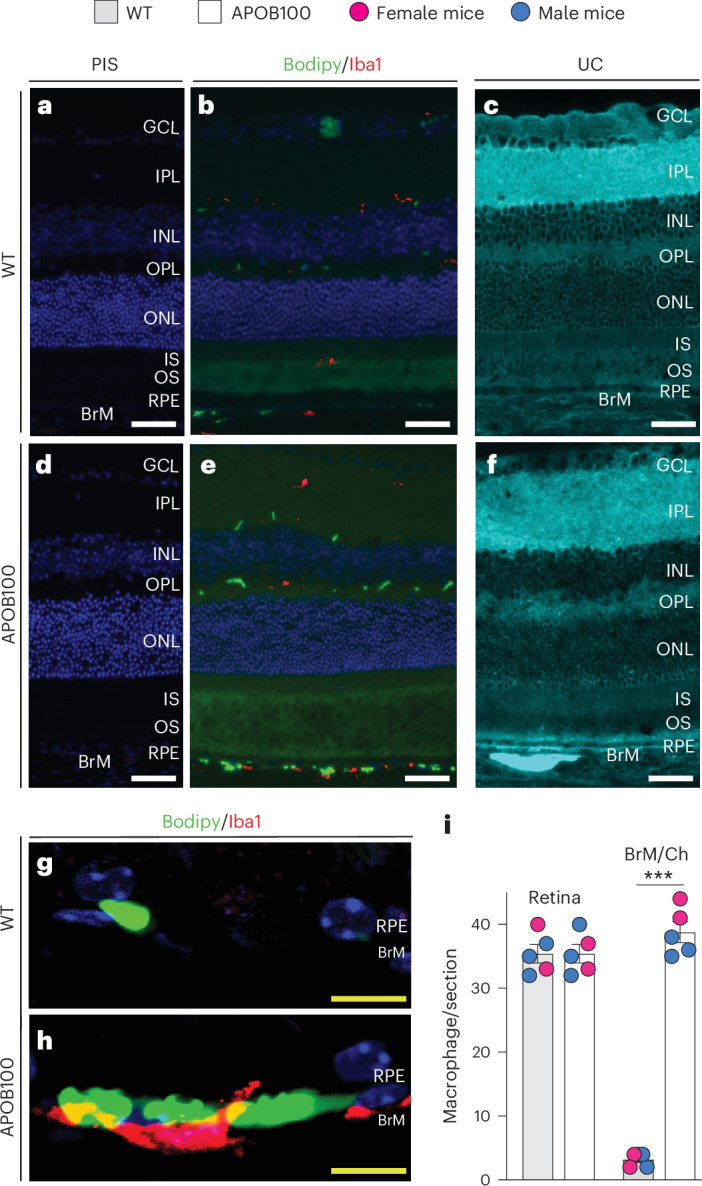
Chorioretinal lipid distribution. **a**–**h**, Representative images (two female and three male mice per genotype) of retinal cross-sections without (**a** and **d**) and with histology stains: Bodipy (green, **b**, **e**, **g** and **h**) and filipin (cyan, **c** and **f**). These sections were also used for staining either with pre-immune serum ((PIS), **a** and **d**) or anti-Iba1 antibody (red, **b**, **e**, **g** and **h**) to detect macrophages. Macrophage interaction with lipid deposits in WT (**g**) and APOB100 (**h**) retinas. **i**, Macrophage counts per retinal section, each from a different mouse but from the same region close to the optic nerve. Data represent the mean ± s.d. of the measurements in individual mice (two female (magenta circles) and three male (blue circles) mice per genotype) and were analyzed by a two-tailed unpaired Student’s *t*-test. ***P ≤ 0.001. The abbreviations for the retinal layers are as in Figs. 5 and 6. Scale bars, 25 μm (white) and 10 μm (yellow).

**Fig. 8:**
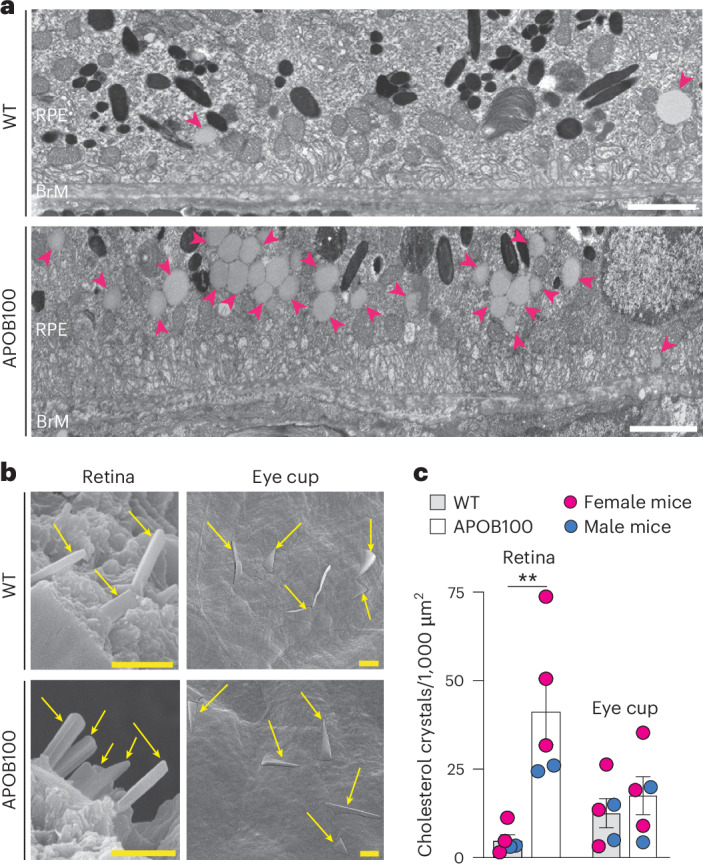
Lipid deposition. **a**, Representative TEM images (four male mice per genotype) of RPE and BrM. Magenta arrowheads indicate lipid droplets. **b**, Representative SEM images (three female and two male mice per genotype) of the retina and eye cup. Yellow arrows point to cholesterol crystals. **c**, Cholesterol crystal quantification. Data represent the mean ± s.e.m. of the measurements in individual mice and were analyzed by a two-tailed unpaired Student’s *t*-test. **P ≤ 0.01. The abbreviations for retinal layers are as in Figs. 5 and 6. Scale bars, 25 μm (white) and 2 μm (yellow).

Two lipid histology stains (Bodipy and filipin) and two types of electron microscopy (transmission electron microscopy (TEM) and scanning electron microscopy (SEM)) were used (Figs. 7 and 8). Stains with Bodipy, which binds to various lipids (UC, EC, TG and free fatty acids)^45^, confirmed lipid accumulation in the form of focal deposits in BrM, which were more numerous in APOB100 than WT mice (Fig. 7a,b,d,e). In addition, focal deposits were detected in the retinal layers containing the plexi of intraretinal vasculature (the ganglion cell layer and the layers above and below the inner nuclear layer), probably reflecting high lipid content in retinal blood vessels of APOB100 mice. The photoreceptor cells were labeled with Bodipy as well but did not have focal lipid deposits.

Stains with filipin, which binds to UC^46^, showed a different labeling pattern as compared with Bodipy. Intense filipin fluorescence was detected at both apical and basal RPE sides of APOB100 but not WT mice (Fig. 7c,f). Thus, at the basal RPE side, a mixture of UC and other lipids was accumulated in the WTD-fed APOB100 mice, consistent with the lipid content of drusen, whereas at the apical RPE side, the accumulations were mainly of UC, consistent with the lipid content of SDDs^8–10,12^.

TEM, which visualizes lipids with different shades of gray when the lipid and membrane preservation is used^47^, revealed clusters of lipid droplets in the RPE of APOB100 mice and isolated lipid droplets in the RPE of WT mice (Fig. 8a). Also, the borders of BrM were poorly defined, especially in APOB100 mice, and their BrM contained numerous electron-dense inclusions of various shapes.

SEM, which enables the detection of cholesterol crystals formed from UC^48^ when organic solvents are not used during sample processing^49,50^, documented cholesterol crystallization in both WT and APOB100 retina (Fig. 8b). Cholesterol crystals were found throughout the retina in ‘cracks’ that appear as a natural process of tissue treatment with osmium tetroxide. Accordingly, it was possible to quantify cholesterol crystals but not to determine their exact retinal location and whether the RPE–photoreceptor interface, which was intensively stained with filipin in APOB100 mice (Fig. 7f), contained more crystals than in WT mice. In any case, our quantifications suggested that there was at least eight times more cholesterol crystals in the APOB100 retina than in the WT retina (Fig. 8c). In addition, we imaged the eye cup after removal of the NR with attached RPE and underlying BrM, thus exposing the Ch. In both WT and APOB100 mice, this led to the detection of large cholesterol crystals piercing from the Ch toward the retinal side, a remarkable finding, although the number of crystals seemed to be similar in the two genotypes (Fig. 8c).

This finding gave impetus to Iba1 immunolocalizations on the Bodipy-stained sections as cholesterol crystals are known to be avidly phagocytized by macrophages and induce macrophage activation^49–51^. Indeed, Iba1-positive cells were detected in the retina and BrM of both WT and APOB100 mice (Fig. 7b,e,g,h) and seemed to interact with some lipid deposits in BrM of APOB100 mice (Fig. 7h). In WT mice, more macrophages were detected in the retina than BrM, whereas in APOB100 mice, the macrophage abundance in the two structures was similar, that is, increased relative to the WT BrM (Fig. 7i) and consistent with a higher BrM lipid deposition in this genotype (Fig. 7b,e).

## Discussion

The present work is a continuation of our studies aimed at understanding how plasma lipid profile can affect the retina^21,26,42,52,53^, and why it is not consistently linked to AMD^7,18^. Previously, we and others showed that cholesterol in the retina originates in part from the systemic circulation^42,54,55^ because the LPPs from the Ch can reach the RPE basal side and interact with various receptors on this side^44,54,56–58^. Yet, the effects on the retina of absolute plasma LPP amounts and the LDL-to-HDL ratio—which deliver and remove cholesterol from extrahepatic tissues, respectively^59^—have not been investigated, leaving important questions unanswered. Here, we put WT and APOB100 mice on a WTD for 13 months to extend our previous studies of the same genotypes on ND^26^.

**Fig. 9:**
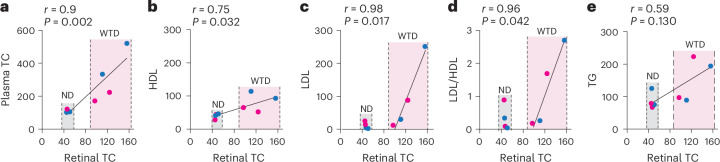
Correlation analyses between the levels of retinal TC and plasma lipids in WT and APOB100 mice. **a**–**e**, For each group, the mean lipid values were taken from Table 1 (blue dots for male mice and magenta dots for female mice). Data were first assessed for normality of distribution by Shapiro–Wilk test. Then, the Pearson correlation test was used for data with normal distribution (plasma HDL (**b**), LDL (**c**) and LDL/HDL (**d**)) and the Spearman test for data that did not pass the normality test (plasma TC (**a**) and TG (**e**)). Data for normal ND and for WTD are highlighted in gray and pink, respectively, and details of these feeding experiments are indicated in Table 1 and the Methods.

Collectively, this and our previous studies enabled a comparison of mean retinal TC levels and plasma lipid profile from all groups of mice studied so far (Table 1). This comparison revealed several correlations: two unconditional, that is, diet-independent, and two conditional, that is, diet-dependent. The unconditional correlations were between retinal TC and plasma TC levels (Fig. 9a) and between retinal TC and plasma HDL levels (Fig. 9b). These correlations suggest that plasma TC and HDL levels should be above certain threshold values to start disturbing retinal cholesterol homeostasis and increase retinal TC levels. The conditional correlations were between retinal TC and plasma LDL levels as well as between retinal TC and the LDL/HDL ratio (Fig. 9c,d), that is, found only in the WTD-fed WT and APOB100 mice (Table 1). These correlations suggest that plasma LDL levels and the LDL/HDL ratio only partially determine retinal uptake of systemic cholesterol, consistent with our previous studies in hamsters^21^. No correlation was found between retinal TC and plasma TG in any group (Fig. 9e). Thus, retinal cholesterol homeostasis could handle increased plasma load of TC, HDL and LDL, probably through compensatory responses^26^, but becomes disturbed when this threshold is exceeded, leading to increased retinal TC levels.

Our correlation analyses raise two questions. First, can the increase in retinal TC levels due to plasma lipid excess lead to AMD in humans as mice do not naturally develop AMD? Second, can the retina-disturbing values of plasma lipids in mice be extrapolated to humans? The present work suggests that, yes, it is certainly possible that cholesterol overload in the retina can lead to the conditions that predispose to AMD. Indeed, the pathognomonic lesions of AMD contain either UC in SDDs, or UC, EC and other lipids in drusen^8–12^. None of these lesions was detected in our WTD-fed APOB100 mice or any currently available mouse models of AMD. Yet, the WTD-fed APOB100 mice had UC deposition at the RPE–photoreceptor interface, the location of UC-rich SDDs (Fig. 7f) plus lipid accumulations below the RPE (Fig. 7e), the location of cholesterol and other lipid-rich drusen. Also, delayed rod-mediated dark adaptation is the first functional risk factor for AMD onset^60–63^, and all scotopic ERG responses were decreased in the WTD-fed APOB100 mice versus WTD-fed WT mice. In addition, there was a decrease in a photopic response (Fig. 5c). Importantly, the amplitudes of all ERG responses were lower than those in the corresponding ND-fed mice^26^. Thus, WTD-fed APOB100 mice model important retinal changes that are prerequisites for AMD better than WTD-fed WT mice, suggesting that retinal cholesterol overload is pathogenic.

With respect to the plasma lipid values that can increase retinal TC levels in humans, these values will probably have to be established clinically because of substantial differences in the human and mouse whole-body cholesterol homeostasis and plasma lipid profile. Perhaps plasma lipid measurements could be included as an additional parameter in the ALSTAR2 study (Alabama Study on Early Age-related Macular Degeneration 2), which longitudinally investigates retinal structure and visual function during the transition from aging to AMD. Participants in this study are already well characterized with respect to their AMD status, retinal function, extent and topography of drusen and SDDs, macular pigment density and other parameters^61–66^. Therefore, these individuals could be assessed for correlations with plasma lipids, with analyses guided by our testable hypothesis that plasma lipids must exceed certain threshold values to elevate retinal cholesterol levels and increase AMD risk.

An important finding of our study is the detection of cholesterol crystals in the retina and Ch of the WTD-fed mice, both APOB100 and WT (Fig. 8b). Clusters of cholesterol crystals beneath the RPE are found in 5–7% of neovascular AMD cases^67^ and are called onion signs^68^. They were first detected by SD-OCT as layered hyperreflective lines^68^ and later were shown to be cholesterol crystals, associated with chronic exudation from type 1 neovascularization and use of cholesterol-lowering medication^67^. Therefore, it was suggested that the onion signs may signify systemic hypercholesterolemia that should be monitored^67^. We did not detect the onion signs on SD-OCT in the WTD-fed mice (Fig. 5a). Rather, we found cholesterol crystals in the Ch perforating through the vessel wall toward BrM and RPE (Fig. 8b). This finding is consistent with a previous histology study showing the so-called cholesterol clefts in the Ch of cholesterol-fed rabbits^67^. Although these clefts exhibited the distinctive morphology of cholesterol crystals^67^, cholesterol was extracted from the tissue during its processing, thus precluding unambiguous cholesterol identification. Our study agrees with this previous work and unambiguously detects cholesterol crystals in the Ch by SEM in mice on WTD.

In humans, showers of cholesterol crystals are released in the systemic circulation due to rupture of atherosclerotic plaques, leading to occlusion of small arteries^48,69–71^. In addition, vascular endothelial and other cell types can produce cholesterol crystals because of hyperlipidemia^72–74^. Yet, irrespective of the origin, cholesterol crystals are pathogenic. First, cholesterol crystals are firm and have sharp edges; hence, they can damage tissues mechanically by perforating them. Second, cholesterol crystals are recognized as foreign bodies^75^ and therefore contribute to inflammation^76^ by activating all three complement pathways and NLRP3 inflammasome and leading to release of pro-inflammatory cytokines^77–80^.

Cholesterol crystals in the onion signs and Ch have been identified and suggested, respectively, a decade ago^67^. Nevertheless, the role of cholesterol crystals in AMD etiology and progression have not yet been investigated as most efforts have been understandably focused on drusen and their association with complement activation and inflammation^81–83^. Moreover, circulating cholesterol crystals are usually lost during blood processing for lipid tests and therefore do not affect the blood TC, LDL and HDL cholesterol levels. The present work brings attention to cholesterol crystals in the Ch and provides a model for investigating the cholesterol crystal pathogenicity in the background of hypercholesterolemia but without the confounding contribution of drusen. Previously, we detected cholesterol crystals in the retina and BrM of diabetic donors and models of diabetic retinopathy and showed that they contribute to retinal inflammation, apoptosis, blood–retina barrier breakdown and vision loss in rodent diabetic models^50^. We suggested that cholesterol crystal formation is a unifying pathogenic mechanism in the development of diabetic retinopathy^50^. Here, we describe a mouse model with cholesterol crystals in the choroid and retinal changes that are prerequisites for AMD, allowing us to begin investigating in more detail the role of cholesterol crystals in AMD.

Remarkably, the localizations of cholesterol crystals in diabetic NR and NR of the WTD-fed mice seemed to be different. In diabetic retina, cholesterol crystals were mainly found on the retinal surface, at the RPE–photoreceptor interface and in BrM^50^. In our mice, cholesterol crystals were found within every retinal layer and were not abundant on retinal surface. This difference suggests that the mechanisms that underlie retinal cholesterol crystallization are perhaps not the same in the two disease models, although they can overlap, due to differences between diabetes and AMD in retinal cholesterol dyshomeostases, systemic lipid profiles and disruption of the blood–retinal barriers^7,18,41,84,85^. As a result, the pathological consequences in the two diseases are different, as exemplified by a different pattern of neovascularization: from the retinal surface in the vitreous in diabetic retinopathy and from the Ch in the retina or from retina in the Ch in AMD. Nevertheless, both disease models underscore the importance of cholesterol crystals in the Ch and measurements of circulating cholesterol crystals either directly or indirectly by determining plasma levels of Lp(a), a cholesterol-rich LDL-like particle with one molecule of APOB100 covalently bound to a molecule of apolipoprotein (a)^59^. Indeed, the Lp(a) levels are primarily influenced by genetics^73,86–88^ and were considerably different in patients with acute coronary syndrome presenting with or without cholesterol crystals in the culprit lesion, although the patients had similar baseline clinical characteristics, including plasma TC, LDL and HDL^88^. Perhaps the Lp(a) levels should also be investigated for a link to AMD.

Lastly, in the WTD-fed WT and WTD-fed APOB100 mice, HDL_3_ heterogeneity was increased with the density of the additional HDL_3_ subpopulations being different in the two genotypes: it was higher in WT mice (HDL_3-3_ and HDL_3-4_) and lower in APOB100 mice (HDL_3-2_; Fig. 2b). As HDL density is increased when there is a decrease in the HDL cholesterol content^89^, it is likely that less cholesterol was present in the WT HDL_3-3_ and HDL_3-4_ than in the APOB100 HDL_3-2_, reflecting the lower plasma TC content in WT mice (Table 1). This difference in densities of the additional HDL_3_ subpopulations also suggests that WT HDL_3-3_ and HDL_3-4_ could promote cellular cholesterol efflux more efficiency than APOB100 HDL_3-2_ as this was shown for HDL_3_ versus HDL_2_ (refs. ^35,36,90^). In addition, HDL was found to dissolve cholesterol crystals in vitro and human atherosclerotic plaques^91^, although different HDL subclasses and subpopulations within each subclass have not yet been compared for their ability to dissolve cholesterol crystals. Thus, increased HDL_3_ heterogeneity in the WTD-fed animals is probably a compensatory response to the increased need of cholesterol removal from extrahepatic tissues due to increased tissue cholesterol supply by LDL. Also, it could be a mechanism to dissolve cholesterol crystals in systemic vasculature. Future studies are clearly needed to elucidate the roles of HDL heterogeneity in both tissue cholesterol efflux and the dissolution of vascular cholesterol crystals.

In summary, feeding WT and APOB100 mice a WTD for 13 months led to systemic hyperlipidemia in both genotypes, but of differing severity, and altered HDL_3_ heterogeneity. The WTD-fed APOB100 mice developed a human-like hyperlipidemia and had higher increases in retinal cholesterol uptake from the systemic circulation and retinal TC levels compared with WTD-fed WT mice. They also had reductions in retinal function and increases in lipid deposition above and below the RPE, cholesterol crystallization in the retina, and subRPE macrophage infiltration. Remarkably, both genotypes had cholesterol crystals in the Ch, with their tips piercing through the blood vessels and pointing toward BrM. Thus, the WTD-fed APOB100 mice represent a model of human-like hyperlipidemia, cholesterol crystallization in the Ch and retinal changes prerequisite for AMD. Our data suggest potential links between plasma lipid profiles and AMD, and indicate that cholesterol crystals in the systemic circulation and choroid warrant further investigation as contributors to AMD development and progression.

## Methods

### Animals

APOB100 mice on the C57BL/6N background were from Taconic Biosciences (no. 1004). These mice had the retinal degeneration *Crbl*^*rd8*^ mutation, which was bred out from our colony by crosses with mutation-free C57BL/6J mice from The Jackson Laboratory (no. 000664). Only hemizygous APOB100 Tg mice were used with nontransgenic littermates serving as WT. At about 1 month of age (right after weaning), all animals were put on WTD (TD.88137 Envigo, now Inotiv), containing 0.15% (w/w) cholesterol and 21% (w/w) milk fat. This dietary treatment continued for 13 months (until the age of 14 months), during which mice were maintained on a standard 12-h light (~10 lux)–dark cycle with WTD and water provided ad libitum. In our previous studies, ND was used (Prolab Isopro RMH 3000), which contained 0.02% (w/w) cholesterol and 5% (w/w) fat and which was given to mice for 11 months from weaning to 12 months of age^26^. All animals were housed in the animal facility at Case Western Reserve University under the supervision of trained personnel. This facility conforms to all state and federal guidelines concerning the care and treatment of experimental animals. For in vivo evaluations, mice were anesthetized via intraperitoneal injection with 80 mg/kg ketamine and 7 mg/kg xylazine. For experiments that required retina or eye isolation, cervical dislocation was performed. All animal experiments and euthanasia methods were approved by Case Western Reserve University’s Institutional Animal Care and Use Committee and conformed to recommendations of the Panel on Euthanasia of the American Veterinary Medical Association (protocol 2014-0154).

### Retinal structure and function

Retinal gross structure and thickness of retinal layers were assessed by SD-OCT, which was carried out as described^93^, using an Envisu R2200 UHR OCT imaging system (Leica Bioptigen). Retinal function was evaluated by ERG responses under scotopic or dark-adapted conditions (a- and b-waves) or photopic or light-adapted conditions (b-waves). These responses were elicited by strobe flash stimuli as described^94^. To record the photopic c-wave, standard full-field stimulators were positioned on each eye, and a stimulus of 150 cd s/m^2^ luminance lasting for 100 ms was applied. c-wave responses were measured for 5 s following stimulus onset. The c-wave amplitude was quantified as the difference between the baseline (0 µV) and the most positive peak occurring between 500 ms and 3500 ms after stimulus onset. All ERG responses were recorded with the Celeris Model D430 system (Diagnosys LLC).

### Status of vascular networks

Intraretinal and choroidal vascular networks were evaluated by FA and ICGA, respectively, as described^26^, using a scanning laser ophthalmoscope (Spectralis HRA, Heidelberg Engineering). For FA, mice received a bolus (0.1 mL) intraperitoneal injection of 1.0% sodium fluorescein (Akorn, no. 17478-250-20) in phosphate-buffered saline (PBS). For ICGA, mice were given a bolus tail vein injection of ICG (Diagnostic Green, NDC 70100-424-01), 2 mg/kg of body weight or ~0.02 mL of the 2.5 mg/mL solution in sterile water. ICGA can also detect lipid accumulations in BrM as they impede the dye passage from the Ch to the RPE and are presented as hypofluorescent spots on late phase ICGA^95–100^. Conversely, the ICG fundus fluorescence is homogeneous when there are no lipid deposits in BrM^96,100–102^.

### Chorioretinal LPP trafficking

In the blood, anionic ICG binds to various circulating proteins as well as lipids on LPPs: polar (phospholipids) but not neutral (EC, UC and TG) lipids^103–107^. Hence, the eyes of the ICG-injected mice were used for the visualization of chorioretinal LPP traffic as described^21,26^. Eyes were excised and fixed for 15 min at room temperature in a solution containing 20% dimethylsulfoxide (Fisher Scientific, BP231-100) and 2% paraformaldehyde in PBS. Eyes were then embedded in the Tissue Tek O.C.T. compound (Sakura Finetek USA, no. 4583) and were flash frozen in liquid nitrogen. The retinas were sectioned 15 μm thick and imaged on a Zeiss Axio Scan.Z1 slide scanner (Carl Zeiss Microscopy) equipped with a Colibri 7 far-red light-emitting diode light source and a Plan-Apochromat 20×/0.8 numerical aperture objective. The acquisition software was Zen Blue 3.1. The excitation and emission wavelengths for ICG imaging were 720–750 nm and 769–807 nm, respectively, and 370–400 nm and 412–438 nm, respectively, for DAPI. Images were deconvoluted by the Huygens Essential 23.10 software (Scientific Volume Imaging) using the standard deconvolution profile in Deconvolution Express and represented a maximum intensity projection of the deconvolved *z*-stack. For the fluorescence intensity quantification, Metamorph Imaging Software (Molecular Devices) was used. Retinal cross-sections were randomly selected from mice, and their fluorescence intensity was recorded and averaged for each genotype.

### Plasma lipid and albumin content

Blood was collected via cardiac puncture after mice were fasted overnight and deeply anesthetized the following morning with a bolus injection of 80 mg/kg ketamine plus 7 mg/kg xylazine (Patterson Veterinary, 07-890-8598 and 07-808-1947, respectively). The blood was processed as described to obtain plasma^26^, and aliquots of plasma were sent to IDEXX Laboratories for direct measurements of the LDL, HDL, TC, TG and albumin content.

To isolate LDL and HDL, a separate group of mice was given the ICG injection as described above for ICGA and was euthanized 13 min after injection. Plasma was obtained and subjected to centrifugation at 15,000*g*, 4 °C, for 30 min to remove the chylomicron and light-density LDL fractions, followed by three sequential density ultracentrifugations using KBr as described^21^. In brief, LDL was concentrated at a KBr density of 1.063 g/mL, after which HDL was isolated using density ranges of 1.063–1.125 g/mL (HDL_2_) and 1.125–1.210 g/mL (HDL_3_). After each ultracentrifugation, 1-mL aliquots were collected from the tube top to the tube bottom, and their fluorescence and light scattering were measured to detect LPPs.

### Retinal sterol profile and total retinal cholesterol input

After mouse euthanasia, the retina was isolated and processed as described^94,108^. Retinal sterols were quantified by isotope dilution gas chromatography–mass spectroscopy using deuterated sterols analogs as internal standards. Both TC and UC were measured to calculate the content of EC, which represented the difference between TC and UC.

Total retinal cholesterol input, the sum of in situ biosynthesis and uptake from the systemic circulation, was quantified as described^42^ based on in vivo labeling of the whole-body cholesterol with deuterium (^2^H or D) atoms from deuterated water (D_2_O) as ~22 water molecules are used to synthesize one molecule of cholesterol^109^. This enabled the measurement of the [^2^H] incorporation into retinal cholesterol, and the calculation of the total retinal cholesterol input based on the measurements of the retinal TC levels. Specifically, mice were injected intraperitoneally with 0.5–0.8 mL of D_2_O equal to ~3.5% of mouse body water and were put on 6% D_2_O for 14 days before the end of dietary treatment. Mice were then fasted overnight and deeply anesthetized, and their blood was withdrawn via cardiac puncture. Mice were perfused through the heart with PBS, 50 mL at a 1.5 mL/min flow rate, and their eyes were excised and dissected to obtain the retina (NR + RPE). Tissue processing and calculations of total retinal cholesterol input were as described^42,110^ and normalized to the whole-body [^2^H] water enrichment. The latter was measured after the isotopic exchange with acetone of the serum of mice, which received D_2_O (ref. ^42^).

### Retinal mRNA quantification

Total RNA (1 μg) was isolated using the RNeasy mini kit (Qiagen, no. 74194) and converted to cDNA by SuperScript III Reverse Transcriptase (Thermo Fisher Scientific, no. 18080093). Quantitative reverse transcription polymerase chain reaction was performed in triplicate on an LightCycler 480 instrument (Roche Life Science) and normalized to *Gapdh*. Changes in relative mRNA levels were calculated by the 2^−ΔΔCt^ method^111^.

### Retinal histo- and immunohistochemistry

Retinal lipids were stained as described^46,49,58,93^ with filipin (Cayman Chemical, no. 70440), which binds to UC^46^, and with BODIPY 493/503 (Thermo Fisher Scientific, D3922), which labels UC, EC, TG and free fatty acids^45^.

Retinal immunolocalization of Iba1, a macrophage/microglia-specific protein^112^, was as described^94^, using rabbit polyclonal anti-Iba1 antibody (Wako, catalog no. 019-1974; 1:250 dilution) and Alexa Fluor 647-conjugated goat anti-rabbit IgG (Jackson ImmunoResearch, catalog no. 111-605-144; 1:200 dilution).

### TEM

The preparation of the retina was as described^113^, using the osmium-tannic acid-*para*-phenylenediamine technique to preserve membranes and neutral lipids^47^. The retina was imaged using a 1200EX transmission electron microscope (JEOL).

### SEM

The eye and retinal processing were as described^49,50^, omitting tissue dehydration in the solutions of aqueous ethanol and only using retinal vapor fixation with 2% osmium tetroxide. Retinal samples were analyzed by a JEOL 6610LV (tungsten hairpin emitter) scanning electron microscope (JEOL) to detect cholesterol crystals^48^.

### Statistics

No statistical methods were used to predetermine sample size, which was based on previous experience, and the investigators were not blinded with respect to the mouse genotype. In the nonquantitative evaluations, such as histo- and immunohistochemistry and electron microscopy, retinal regions in WT and APOB100 mice were matched by sex and location. In the quantitative studies, all data were used, and apparent outliers were not excluded. All data were checked for normality using the Shapiro–Wilk test and are presented as mean ± s.d. ERG responses are presented as mean ± s.e.m.; sample sizes are indicated in each figure legend. The data were analyzed either by one- or two-way analysis of variance (ANOVA) with Tukey’s multiple comparison test, an unpaired nonparametric Mann–Whitney test or a two-tailed unpaired Student’s *t*-test using GraphPad Prism software. Statistical significance was defined as *P ≤ 0.05, **P ≤ 0.01, and ***P ≤ 0.001.

### Reporting summary

Further information on research design is available in the Nature Portfolio Reporting Summary linked to this article.

## Online content

Any methods, additional references, Nature Portfolio reporting summaries, source data, extended data, supplementary information, acknowledgements, peer review information; details of author contributions and competing interests; and statements of data and code availability are available at 10.1038/s41684-026-01693-x.

## Supplementary information

Supplementary Information Supplementary Fig. 1.

Reporting Summary

## Data Availability

The data that support the findings of this study are available from the corresponding author upon request.
