# Supplementary material for: Retinal phenotype of APOB100 transgenic mice on a Western diet with human-like hyperlipidemia and cholesterol crystals in the retina and choroid
Source: Lab Anim (NY). 2026 Feb 9;55(3):83–94. doi: 10.1038/s41684-026-01693-x (PMC12956585; doi:10.1038/s41684-026-01693-x)
Supplement: Supplementary file 1 — Supplementary Fig. 1. [file 41684_2026_1693_MOESM1_ESM.pdf]

lab animal

Supplementary information

https://doi.org/10.1038/s41684-026-01693-x

Retinal phenotype of APOB100 transgenic mice on a Western diet with human-like hyperlipidemia and cholesterol crystals in the retina and choroid

In the format provided by the authors and unedited

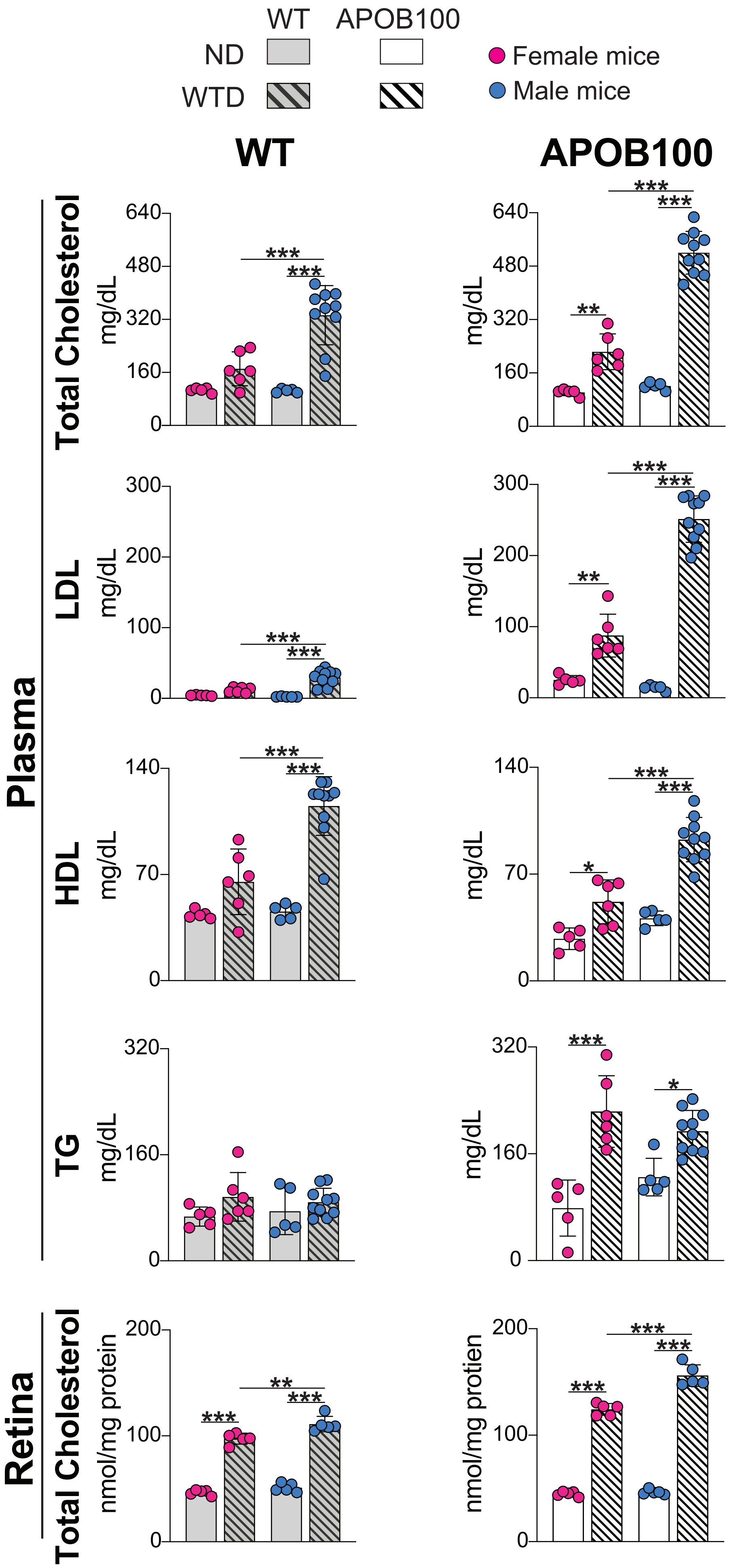

Supplementary Figure 1. A comparision of plasma lipids and retinal cholesterol in mice within the same genotype (WT and APOB100) but on different diets: normal (ND) and Western type (WTD). Data represent the mean ± SD of the measurements in individual animals (n=5-10 per group). *P* ≤ 0.05; ***P* ≤ 0.01; ****P* ≤ 0.01. Data represent the mean ± SD and were assessed by one-way ANOVA with Tukey's multiple comparison test.
